# Estimation of regional irrigation water requirements and water balance in Xinjiang, China during 1995–2017

**DOI:** 10.7717/peerj.8243

**Published:** 2020-01-03

**Authors:** Yinbo Li, Hongwei Wang, Yun Chen, Mingjiang Deng, Qian Li, Adiliai Wufu, Dan Wang, Ligang Ma

**Affiliations:** 1 College of Resource and Environmental Science, Xinjiang University, Urumqi, Xinjiang, China; 2 General College Key Laboratory of Smart City and Environmental Modeling, Xinjiang University, Urumqi, Xinjiang, China; 3 CSIRO Land and Water, Canberra, Australia

**Keywords:** Water requirement, Water balance, Irrigation, Junggar Basin, Tarim Basin

## Abstract

Estimating water requirements and water balance for irrigated agricultural areas are important and will facilitate the efficient allocation of water resources for agriculture while minimizing the impact on natural ecosystems in arid regions. Based on the Penman-Monteith formula and GIS technology, the irrigation water requirements (IWR) of three main crops (cotton, corn and wheat) during the growing season were estimated and their spatio-temporal changes over the past 23 years (1995–2017) were analyzed in Xinjiang province, China. Our results indicated a dramatic increase in IWR from 14.12 billion m^3^ in 1995 to 38.99 billion m^3^ in 2017 due to the rapid cropland expansion of approximately 2.58 × 10^4^ km^2^ in this period. Monthly IWR usually peaked in summer from May to July and varied in different basins. From the perspective of crops, cotton was identified to have consumed the largest amount of water, reaching 26.39 billion m^3^ in 2017, accounting for 67.68% of total water consumption. Spatially, the fastest increasing rate of IWR was Tarim Basin, which was attributable to the increase in water requirement of cotton. By comparing IWR and actual irrigation of Xinjiang in 2014, the amount of water scarcity had reached −15.01 billion m^3^ (−9.80 billion m^3^ in Tarim Basin and −6.58 billion m^3^ in Junggar Basin). The planting areas of three main crops (wheat, corn and cotton) were more sensitive to IWR than rising temperature indicated by our model. This study is of great significance for the scientific allocation of water resources in the irrigated areas of the different prefectures of Xinjiang.

## Introduction

The increase in global temperature has led to widespread decline in water restoration on continents during the past century (Huang et al., 2017; Ji et al., 2014). For example, during the period of 2002–2016, global endorheic systems had an extensive water decrease of approximately 106.3 Gt yr^−1^, being attributable to decrease in surface water, soil moisture and groundwater (Wang et al., 2018b). In addition to burgeoning industrialization, rapid urbanization and fast population growth, the need for water resources is consistently increasing (Cai et al., 2016; Fader et al., 2015; Meng et al., 2019). This situation results in serious pressure on regional water resources (Shteiwi & Raggad, 2018), especially in areas of extreme water shortage. Water for agriculture and industry are the two predominant factors accounting for 54% and 28% in Beijing, respectively (Ye et al., 2018), and accounting for 94.4% and 2.2% in Xinjiang, respectively (Xinjiang Water Resources Bulletin, 2016). According to the China Water Resources Bulletin, the total water withdrawal for agriculture in China is 5.20 × 10^11^ m^3^ in 1993 and increases to 6.04 × 10^11^ m^3^ in 2017. Approximately 90% of the total water withdrawal is utilized for agriculture (Fang, Chen & Li, 2018).

In the current context of declining water reserves, water usage for agricultural irrigation is threatened, which in turn affects crop yields and human food security (Kang et al., 2017; Patil & Tiwari, 2018; Porkka et al., 2016). In China, this situation is even more pronounced in its arid northwest region (e.g., Xinjiang Province), where the environment is extremely harsh and water resources are extremely scarce (Feike et al., 2017). The agricultural planting zone is mainly distributed in the oasis areas of this province, which require irrigation of more than 90% of crops (Song et al., 2018). It is one of the most stressed areas in terms of water resources in northwest China, and has a large area of cultivated land and is also a major producer of grain (Li et al., 2017b). The crops primarily include cotton, wheat and corn, all of which rely heavily on seasonal irrigation. Surface water irrigation (rivers and lakes) is the most important method and accounts for almost 95% of the irrigated area (Li, Qian & Wu, 2018). Long-term excessive water use for agricultural irrigation leads to decreasing water for ecological stability and to accelerating regional ecological crises (Chen et al., 2016; Fang et al., 2018; Li et al., 2017b).

Arid regions such as Xinjiang play a significant role in the global ecosystem and the climate system, and are an important ecological barrier and strategic base of energy and mineral resources in China. Xinjiang is not only an important ecological barrier and strategic base of energy and mineral resources but is also the core area of the “silk road economic belt” proposed by the “One Belt and One Road” initiative (Chen et al., 2016). The construction of an ecological civilization and protection of the ecological environment (especially the water environment) have become major strategic goals in China because of a key guarantee for sustainable and healthy economic development (Sun et al., 2018b). Due to the shortage of water resources in Xinjiang, the need for rationally allocating its limited water resources (Loucks, 2017) has become an increasingly urgent issue. Since agricultural irrigation in Xinjiang is the most important water usage, determining water consumption of different crops in Xinjiang should be given priority. Several works regarding the water consumption of crops in Xinjiang have been conducted by scientists, including the irrigation methods (Feike et al., 2017) and water demand (Guo et al., 2015; Shen et al., 2013; Wang et al., 2019). However, their results overestimated the spatial demand of irrigation water because of the single crop coefficients that only came from the Yanqi station in whole Xinjiang. The crop coefficients should be different in different parts of Xinjiang due to rapid changes in climate and heterogeneousness of landscape. In addition, most studies focusing on IWR were limited in spatial scale, such as in Tarim or Junggar Basin (Shen et al., 2013). Therefore, the gap of IWR estimation in both large spatial and temporal scales of Xinjiang need to be filled.

The changing climate (temperature and precipitation) will directly affect agricultural IWR. Studies have analyzed the influence of climate change on IWR and have estimated the available water amounts and IWR under future climate change scenarios (Fan et al., 2016; Liu et al., 2017; Zhou et al., 2017). For example, affected by rising temperatures, IWR in the arid northwest areas will increase by approximately 4.27–6.15 billion m^3^ over the next 60 years (Guo et al., 2015; Guo & Shen, 2016; Sun et al., 2018a). To better serve crop irrigation practices and scientific allocation and utilization of water resources in different regions of Xinjiang, the following four issues are addressed in this study: (1) Can the spatial variations of IWR in Xinjiang be mapped from 1995 to 2017? (2) How does IWR change in annual and monthly scales in different basins (Junggar Basin, Yili Basin, Turpan-Hami Basin and Tarim Basin) over the past 23 years? (3) How does the IWR vary with respect to three main crops (i.e., wheat, corn and cotton) across four basins and along 23-year-time scale? (4) Are there any relationships between IWR and arable land area as well as temperature?

## Studied Area and Methods

### Study area

**Figure 1:**
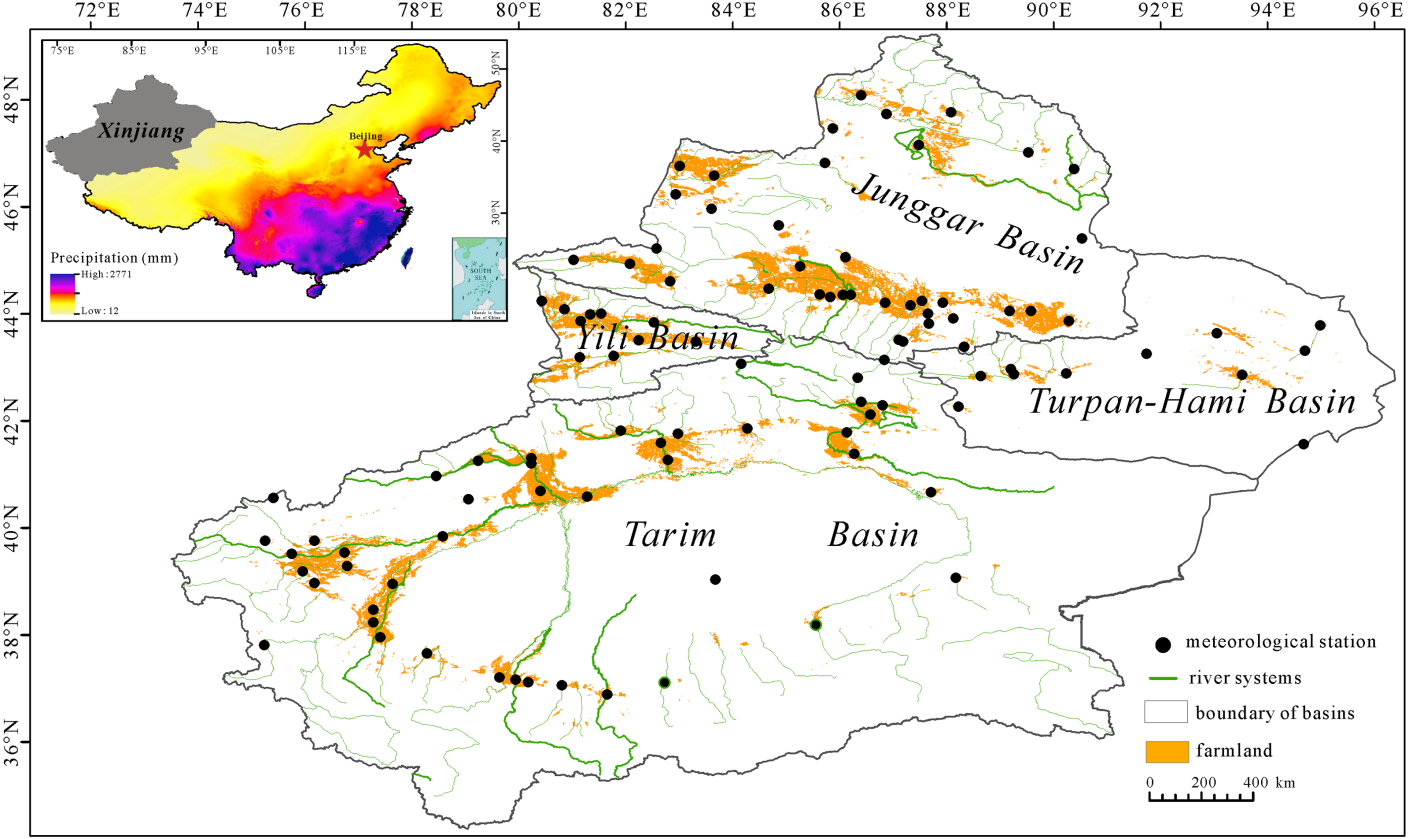
Distribution of croplands, meteorological stations and river systems in Xinjiang within China.

Xinjiang (34°–48°N, 73°–96°E) is located in the northwestern part of China (Fig. 1). It covers an area of approximately 166 × 10^4^ km^2^ and accounts for 1/6 of the total land area in China. The area has a temperate continental climate with an average annual precipitation of less than 200 mm and an average annual temperature of approximately 9.1 °C. Three mountain ranges (the Altai Mountains, the Tianshan Mountains and the Kunlun Mountains) are sandwiched between two basins (Junggar Basin and Tarim Basin) and are the most characteristic geomorphic features in Xinjiang. The oases are mainly situated in the piedmont plains and their water resources primarily result from rivers originating from precipitation and from glacial and snow melt water in the mountainous regions. All cultivated lands are mainly limited to the oasis regions embedded at the edges of the deserts; wheat, cotton and corn are the main crops relying on irrigation from surface water and groundwater.

**Figure 2:**
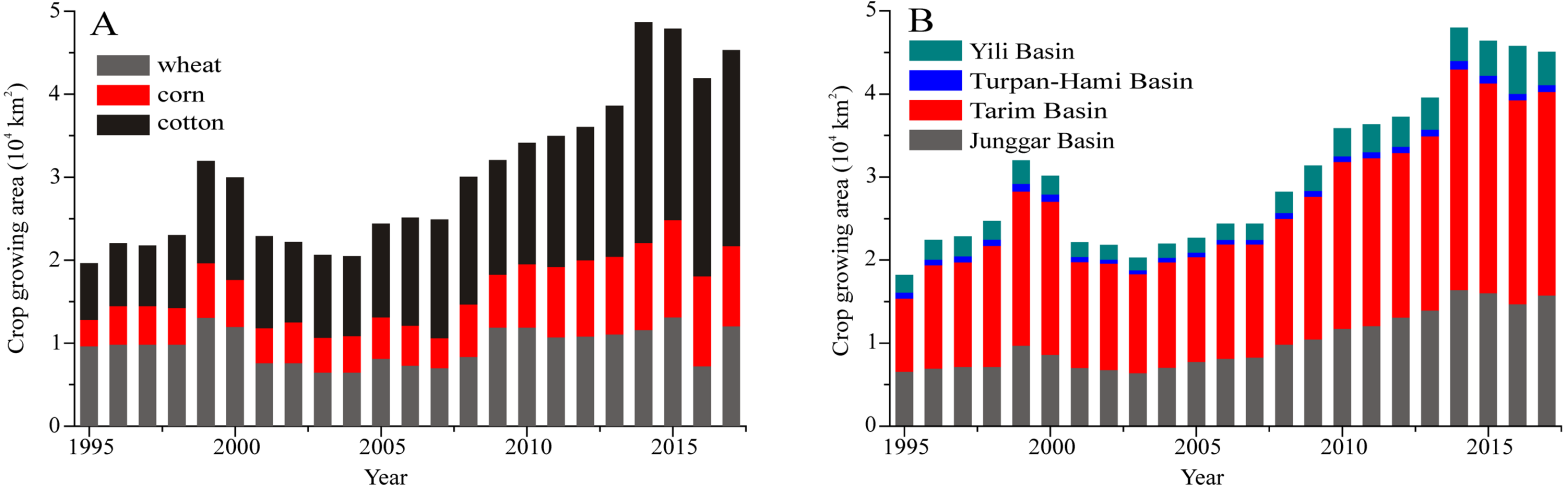
Changes in crop growing areas for different crops (A) and for different basins (B).

By the end of 2017, the crop growing area had reached 4.53 × 10^4^ km^2^ in Xinjiang (Fig. 2A). Cotton growing area was the largest making 2.36 × 10^4^ km^2^, accounting for almost 52% of all crop growing area. Wheat growing area made up about 25% of the total crop area, with corn growing area in third place with 22.4%. Compared with the growing area in 1995 (1.95 × 10^4^ km^2^), the total growing area in Xinjiang expanded by 2.32 times. The expansion of cotton growing areas was the fastest spanning from 0.68 × 10^4^ to 2.36 × 10^4^ km^2^. The wheat growing area increased by approximately 0.25 × 10^4^ km^2^, and the corn growing area increased by approximately 0.64 × 10^4^ km^2^. The cotton acreage exhibited the most rapid increase in Tarim Basin from just 0.49 × 10^4^ km^2^ in 1989 to 1.49 × 10^4^ km^2^ in 2017 (Fig. 2B). The maximum crop growing area during the studied interval occurred in 2014 (4.76 × 10^4^ km^2^) and in 2015 (4.66 × 10^4^ km^2^), and exhibited a slight decline in the following two years. According to the statistics of the Xinjiang Water Resources Department (2014, 2015), the irrigation withdrawal volume increased to 29.30 billion m^3^ in 2014 and 28.66 billion m^3^ in 2015.

### Data

Based on the topography, Xinjiang is classified into four basins (Junggar Basin, Yili Basin, Turpan-Hami Basin and Tarim Basin) (Fig. 1) and this region is composed of 15 prefectures including Urumqi, Changji, Shihezi, Karamay, Turpan, Hami, Aletai, Tacheng, Bozhou, Yili, Aksu, Bazhou, Kezhou, Hotan and Kashgar. The administrative boundaries were downloaded from the website (http://www.resdc.cn/).

The 66 related meteorological datasets for Xinjiang were collected from the China Meteorological Administration (http://data.cma.cn/), including the maximum temperature, the minimum temperature, mean monthly temperature, mean monthly precipitation, wind speed, air pressure, relative humidity and sunlight hours.

The land use/cover data for 1995, 2000, 2005, 2010 and 2015 were obtained from the National Land Use/Cover Dataset (NLCD), and were provided by the Earth System Science Data Sharing Infrastructure of the Chinese Academy of Sciences (http://www.resdc.cn). The NLCD maps were generated by visual interpretation of Landsat Thematic Mapper (TM) images. These maps were extrapolated to a resolution of 1 km (Yang et al., 2018).

Socioeconomic data, including crop planting types and areas, were obtained from Xinjiang Statistics Yearbook and Xinjiang Production and Construction Corps Statistics Yearbook and from the Xinjiang National economic and social development statistical bulletin. Irrigation water consumption data for prefecture-level cites in Xinjiang were compiled according to Xinjiang Water Resources Bulletin (2014, 2015). These socioeconomic data were combined with related meteorological data to establish the spatial and temporal characteristics of crop water consumption during 1995–2017 in Xinjiang.

We compared the land use/cover data (1995, 2000, 2005, 2010 and 2015) and crop growing areas from Xinjiang Statistics Yearbook and Xinjiang Production and Construction Corps Statistics Yearbook. The cultivated land cover inferred from the NLCD is greater than that from the data in the Xinjiang Statistics Yearbook and the Xinjiang Production and Construction Corps Statistics Yearbook. Considering the objective of this study focuses on crops while few available remote sensing data can meet that demand. Therefore, data from Xinjiang Statistics Yearbook were adopted in this study. In addition, considering the objective of this study focuses on different time-scale and spatial-scale features of crops and related irrigation water demand, widely accepted grid NLCD data were selected to consider as the boundary of crop growing area.

### Methods

To calculate IWR for different crops (wheat, corn and cotton), we need to calculate parameters such as evapotranspiration (ET_0_), crop coefficients during the growing interval of crops (*K*_*c*_), effective rainfall (*P*_*e*_), crop water requirement (CWR) and irrigation coefficients (*I*_*e*_) by meteorological factors (Allan, 2003; Allen et al., 1998; Cammarano et al., 2016). In this study, based on the meteorological data from 66 stations, spatial interpolation of climate factors was carried out by the Thiessen polygon method in ArcGIS 10.4. The detailed methods for calculating these parameters are described below.

#### References evapotranspiration (ET_0_)

According to the Penman-Monteith equation, we calculated the reference evapotranspiration (ET_0_) (Allen et al., 1998; Allan, 2003), and ET_0_ was calculated using the following equation:

(1) \begin{eqnarray*}& & {\mathrm{ET}}_{0}= \frac{0.408\Delta \left( {R}_{n-}G \right) +y \frac{900}{T+273} {u}_{2} \left( {e}_{s}-{e}_{a} \right) }{\Delta +y \left( 1+0.34{u}_{2} \right) } \end{eqnarray*}

where ET_0_ is the reference evapotranspiration (mm/d), *R*_*n*_ is the net surface radiation [MJ/(m^2^ d)], G is the soil heat flux density [MJ/(m^2^ d)], *u*_2_ is the wind speed at a height of 2 m (m/s), *e*_*s*_ is the saturated vapor pressure (kPa), *e*_*a*_ is the actual vapor pressure (kPa), Δ is the slope of vapor pressure–temperature curve (kPa), and y is the psychrometric constant (kPa).

#### Crop coefficient (*K*_*c*_)

The *K*_*c*_ values for four stages of cotton, wheat and corn were calculated by the FAO single crop coefficient method. Growth stages include the starting point of the growth cycle, the division of the initial growth period, rapid development period, mid-maternity period, and mature period. FAO has established standard values of *K*_*c*_ at different stages in the early, middle, and maturation of crop coefficients (*K*_cini_, *K*_cmid_ and *K*_cend_) and is based on related meteorological and soil conditions. In the early stage of crop growth, *K*_cini_ values are mainly based on FAO. *K*_cmid_ values in the middle stage of crop growth are calculated according to the following formula:

(2) \begin{eqnarray*}{K}_{\mathrm{cmid}}={K}_{\mathrm{cmid(s)}}+ \left[ 0.04 \left( {u}_{2}-2 \right) -0.004 \left( {\mathrm{RH}}_{\mathrm{min}}-45 \right) \right] { \left( \frac{h}{3} \right) }^{0.3}\end{eqnarray*}

where *K*_cmid(s)_ is the standard value from FAO, RH_min_ is the minimum relative humidity, and *H* is the crop height (Wang et al., 2018a).

In the late growth stage, *K*_cend_ is calculated by:

(3) \begin{eqnarray*}{K}_{\mathrm{cend}}={K}_{\mathrm{cend(Tab)}}+ \left[ 0.04 \left( {u}_{2}-2 \right) -0.004 \left( R{H}_{\mathrm{min}}-45 \right) \right] { \left( \frac{h}{3} \right) }^{0.3}\end{eqnarray*}

where *K*_cend(Tab)_ is from the FAO recommended standard values, and RH_min_ is calculated as follows:

(4) \begin{eqnarray*}{\mathrm{RH}}_{\mathrm{min}}= \frac{{e}^{o}{T}_{\mathrm{min}}}{{e}^{o}{T}_{\mathrm{max}}} \times 100\text{%}\end{eqnarray*}

where *T*_min_ and *T*_max_ are the minimum and maximum temperature, respectively, and *e*^*o*^ is the saturated vapor pressure at the corresponding temperature.

#### Effective precipitation (*P*_*e*_)

The effective precipitation for agricultural land is defined as the portion of precipitation that penetrates the canopy and infiltrates into the soil layer and is stored in the crop root zone, usually calculated as follows:

(5) \begin{eqnarray*}& & {P}_{e}=P\times \delta \end{eqnarray*}

where *P* is the precipitation amount, and *δ* is an empirically effective utilization coefficient of precipitation (*δ* = 0.52) (Shen et al., 2013).

#### Crop water requirement (CWR)

The calculation formula for CWR is as follows:

(6) \begin{eqnarray*}& & \mathrm{CWR}={\mathrm{ET}}_{c}-{P}_{e}\end{eqnarray*}

where CWR is the crop water requirement and *P*_*e*_ is the effective precipitation.

In the crop coefficient method, crop evapotranspiration is equal to the reference evapotranspiration (ET_0_) multiplied by the crop coefficient (Kc):

(7) \begin{eqnarray*}& & {\mathrm{ET}}_{c}={K}_{c}\times {\mathrm{ET}}_{0}\end{eqnarray*}

where ET_*c*_ is the crop evapotranspiration, *K*_*c*_ is the crop coefficient, and ET_*o*_ is the reference evapotranspiration.

#### Irrigation efficiency (*I*_*e*_)

The ratio between the net crop water amounts and the total amounts of water withdrawal from river channels or reservoirs is considered as the irrigation efficiency (Wu & Zhou, 2011). The data inferred from the Xinjiang Water Resource Bulletin show a clear increase in *I*_*e*_ from 0.38 in 1990 to 0.68 in 2017. The increase in *I*_*e*_ has resulted from improvements in the irrigation infrastructure as well as the development of water-saving technologies. In this study, the *I*_*e*_ values (Table 1) are used to calculate IWR for the different prefectures of Xinjiang.

**Table 1:** Irrigation efficiency for the different prefectures of Xinjiang.

	**Aksu**	**Aletai**	**Bazhou**	**Bozhou**	**Changji**	**Hami**	**Hotan**	**Kashgar**
*I*_*e*_	0.70	0.62	0.65	0.60	0.70	0.66	0.72	0.63

	**Kezhou**	**Karamay**	**Shihezi**	**Tacheng**	**Turpan**	**Urumqi**	**Yili**	**Xinjiang**
*I*_*e*_	0.60	0.79	0.71	0.69	0.79	0.78	0.73	**0.68**

#### Irrigation water requirement (IWR)

The calculation formula for the crop irrigation water requirement (IWR) is as follows:

(8) \begin{eqnarray*}& & \mathrm{IWR}=\mathrm{A} \mathrm{CWR}/{I}_{e}\end{eqnarray*}

where A is the planting area of the crop, and CWR is the crop water requirement.

#### Soil water balance

Soil water balance is an indirect method for estimating evapotranspiration and it is based on the principle of conservation of mass:

(9) \begin{eqnarray*}& & {P}_{e}+I+{Q}_{g}-E{T}_{c}-R-D=\Delta W\end{eqnarray*}

where *P*_*e*_ is the effective precipitation per unit area, *I* is the irrigation per unit area, *Q*_*g*_ is the contribution from the water table per unit area, ET_*c*_ is the actual evapotranspiration per unit area, *R* is the surface runoff per unit area, *D* is the deep drainage per unit area and Δ*W* is the change in soil water storage (Zhang et al., 2007). In this equation, R and *D* are neglected as the cultivated area is flat and the precipitation is not intensive. The contribution from *Q*_*g*_ is considered negligible because the water table is often deep in arid Xinjiang region. The soil-water-balance Eq. (9) can be simplified as:

(10) \begin{eqnarray*}& & {P}_{e}+I-{\mathrm{ET}}_{c}=\Delta W\end{eqnarray*}

(11) \begin{eqnarray*}& & I-\mathrm{CWR}=\Delta W.\end{eqnarray*}

The soil-water-balance equation can be further expressed as:

(12) \begin{eqnarray*}& & {I}_{a}-\mathrm{IWR}=\Delta W\end{eqnarray*}

where *I*_*a*_ is the irrigation in a certain area or in a prefecture, IWR is the crop irrigation water requirement in a certain area or in a prefecture.

#### Random forest modeling

To explore the relationships between IWR and crop planting areas and temperatures, we adopted the random forest regression (John et al., 2018) method, which is implemented in the VarlmpPlot package in the R language, to model changes in IWR, the cultivated areas of three crops (wheat, corn and cotton) and temperatures in 15 prefecture levels over 23 years. A total of 345 data points for each crop, 80% of which were modeled and 20% of which were verified. We also analyzed the importance of planting areas and temperature to evaluate the relative contributions of the prediction variables to the modeling process.

It should be noted that the spatial pattern of crops and IWR were derived based on NLCD data to show the relatively high/low value in spatial and all results relating to crop types were based on statistical data from yearbook.

## Results

### References evapotranspiration and precipitation in spatial scale

**Figure 3:**
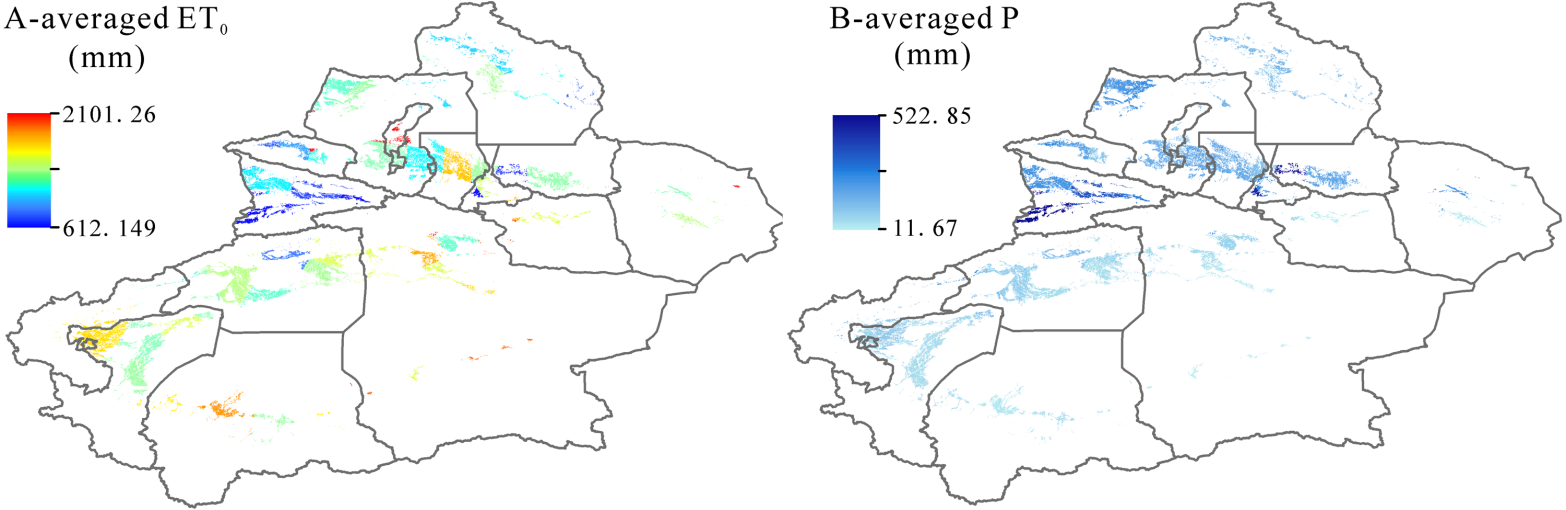
Spatial distribution of averaged references evapotranspiration (ET_0_) (A) and averaged precipitation (P) (B) in Xinjiang during 1995–2017.

Based on the Penman-Monteith equation, we calculated the averaged references evapotranspiration (ET_0_) of Xinjiang during 1995–2017 and the related results were showed in Fig. 3A. The highest ET_0_ was in Tarim Basin including Aksu, Bazhou, Hotan, Kashgar and Kezhou with an average value of 1155.32 mm. The lowest ET_0_ was recorded in Yili Basin with 747.24 mm. ET_0_ in other two basins (Junggar Basin and Turpan-Hami Basin) were 964.74 mm and 1093.09 mm, respectively. It is well known that regional ET_0_ is closely related with precipitation variations (Fig. 3B). In Xinjiang, the lowest-precipitation region was in Tarim Basin with an average value of 41.62 mm, being correspondent with the highest ET_0_ in this region. Similarly, the highest precipitation was occurred in Yili Basin with 340 mm.

### Irrigation water requirement in spatial scale

**Figure 4:**
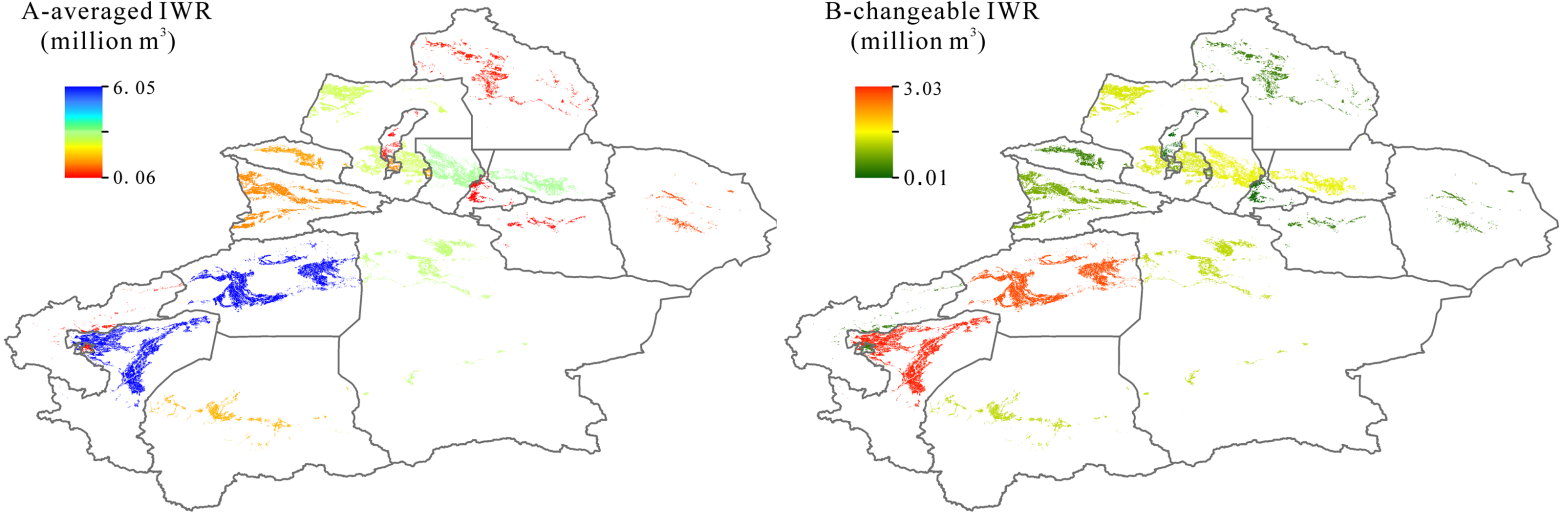
Spatial distribution of averaged irrigation water requirement (IWR) (A) and their changes (B) in Xingjiang during 1995–2017.

Figure 4A shows the averaged states of IWR in 15 prefectures within Xinjiang during 1995-2017. We find the highest IWR in Kashgar and in Aksu within Tarim Basin and their averaged IWR values were 6.05 and 5.00 billion m^3^, respectively. The second highest IWR were found in Bazhou (2.49 billion m^3^) within Tarim Basin and in Tacheng (2.33 billion m^3^) and Changji (2.71 billion m^3^) within Junggar Basin. IWR values in other remaining prefectures (including Urumqi, Karamay, Shihezi, Aletai, Bozhou, Kezhou, Hotan, Yili, Turpan and Hami) were below 2.00 billion m^3^, the minimum IWR were in Urumqi and Karamay, and the IWR values were 0.06 billion m^3^. We also calculated the changeable amounts of IWR in the past 22 years and the results were shown in Fig. 4B.

Being similar to the spatial distributions of averaged IWR, the most rapidly increasing levels of IWR in Kashgar and Aksu within Tarim Basin correspond to the highest averaged IWR values. The IWR in Kashgar increased from 3.03 million m^3^ in 1995 to 6.06 million m^3^ in 2017, while in Aksu, the IWR increased from 2.81 million m^3^ in 1995 to 5.63 million m^3^ in 2017. The same feature of changeable IWR was seen in Junggar Basin. The IWR in Changji increased from 1.40 million m^3^ in 1995 to 2.81 million m^3^ in 2017, while in Tacheng, it increased from 1.32 million m^3^ in 1995 to 2.65 million m^3^ in 2017.

### Irrigation water requirement in temporal scale

**Figure 5:**
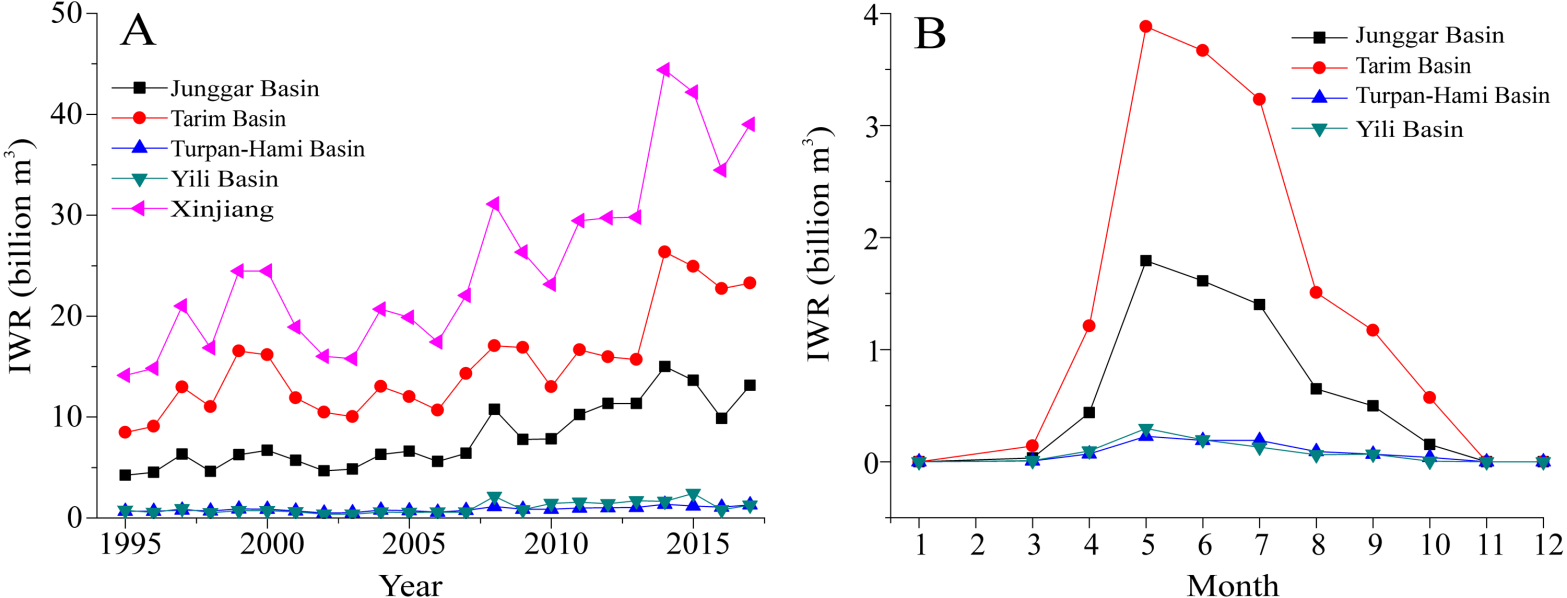
Changeable trends in the IWRs of all crops at an annual scale (A) and at a monthly scale (B) in the different basins of Xinjiang during 1995–2017.

Given the supplies of available water resources in Xinjiang, we pay attention on the IWR variations in four basins. The changeable trends in IWR of different basins during 1995–2017 were presented in Fig. 5A. For four basins, the fastest increase in IWR was recorded in Tarim Basin, from 8.46 billion m^3^ in 1995 to 23.26 billion m^3^ in 2017. Correspondingly, the IWR showed an increase at a rate of 0.41 billion m^3^ each year from 1995-2017. The slight increase was found in Turpan-Hami and Yili Basins in the past 23 years and their rates were 0.03 billion m^3^ per year and 0.05 billion m^3^ per year, respectively. The IWR was highest in 2014, consistent with the crop growing area for that year being the largest in the past two decades. The Tarim Basin shared >60% of the total irrigation water, while the Turpan-Hami Basin only used <5%. The IWR in Tarim Basin was consistently higher than that in Junggar Basin, mainly due to the massive percentage of cotton expansion. Overall, the results revealed that the IWR of Xinjiang rapidly increased from 14.12 billion m^3^ in 1995 to 38.99 billion m^3^ in 2017. The amount of this increase was 24.87 billion m^3^ since 1995. The increase in IWR during the past 23 years was attributable to reclamation, to the increase in crop growing area and to adjustments of the planting structure since 1995.

The monthly variations of IWR in four basins were analyzed and were presented in Fig. 5B. The results showed that definite differences were seen in the monthly IWR of the four basins. In Tarim Basin, the highest IWR were found between May and July. This was attributable to the fact that the planting system for cotton and corn crops in Xinjiang consists mainly of one season. Wheat in Yili Basin gradually develops to the jointing and heading stage in May, and the associated IWR quickly increases and reaches approximately 3.88 billion m^3^ in May. Meanwhile, corn enters the seedling stage in summer. Wheat is harvested in June, the related IWR decreases, leading to the highest value found in this study. After June, the development of summer corn was mainly responsible for IWR in Tarim Basin. In July, cotton is at the flowering stage and corn also meets an important water demand stage. This led to the IWR reaching a maximum of approximately 3.23 billion m^3^ each month. A similar feature of monthly IWR was seen in Junggar Basin, with a lower IWR for every month than that in Tarim Basin. In Turpan-Hami and Yili Basins, the IWR of the local crops per unit area was relatively smaller in comparison with that in Tarim and Junggar Basins. This clear difference was attributed to the relatively smaller planting areas in these two basins (Turpan-Hami and Yili Basins).

### Irrigation water requirements for crops in four basins

**Figure 6:**
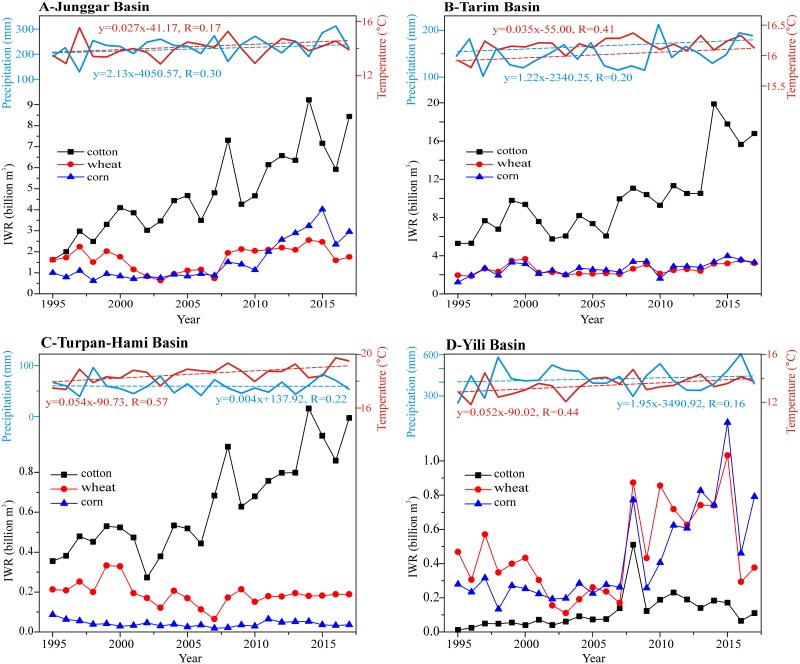
Changeable trends in the IWRs of different crops in the different basins of Xinjiang during 1995–2017. Note: (A) Junggar Basin, (B) Tarim Basin, (C) Turpan-Hami Basin, (D) Yili Basin.

Figure 6 shows changes in IWR for four basins within Xinjiang during 1995–2017. We find that cotton has the highest increasing rate among three crops in three basins (Junggar, Tarim and Turpan-Hami Basins) during the past 23 years (Figs. 6A–6C). Among these three basins, the highest rate of increase of IWR was recorded in Tarim Basin and IWR rapidly increased from 5.29 billion m^3^ in 1995 to 16.77 billion m^3^ in 2017. This was an increase of 11.48 billion m^3^ since 1995. The increase was mainly attributable to the expansion of the planting area for cotton in the studied interval (Fig. 6B). The IWR of other corps (corn and wheat) increased slightly. In Junggar Basin, corn and wheat have shown a strong expansion since 2008. The amount of increase was 0.14 billion m^3^ for wheat and 1.95 billion m^3^ for corn over the past 23 years (Fig. 6A). In contrast changes in IWR for wheat and corn in these two basins (Junggar Basin and Tarim Basin), a decrease in IWR for wheat and corn was found, and the amounts of the decreases were −0.02 billion m^3^ and −0.05 billion m^3^, respectively (Fig. 6C). The increases in IWR for wheat and corn in Yili Basin are similar to those in Junggar Basin. They were both featured by clear increases since 2008 (Fig. 6D). In contrast to the consistent increase in IWR for cotton in Tarim, Junggar and Turpan-Hami Basins, IWR for cotton in Yili Basin was characterized by a slight climbing trend with an increase of approximately 0.10 billion m^3^.

Obviously, the combination of temperature and precipitation changes (Fig. 6) and the changing structure of crop planting (Fig. 2) were mainly responsible for the increase in crop water requirements, which had resulted in a quickly rising rate of IWR in Xinjiang. As shown in Fig. 6, temperature in different basins of Xinjiang had all experienced significant increases and the rates were 0.27 °C/decade in Junggar Basin, 0.35 °C/decade in Tarim Basin, 0.54 °C/decade in Turpan-Hami Basin and 0.52 °C/decade in Yili Basin. However, precipitation changes for all of the above basins were insignificant over the past 23 years. Based on the above analysis, we propose that increases in IWR in different basins of Xinjiang were attributable to changes in crop growing areas and that these are closely related to changes in planting structure. The increase in cotton growing area was mainly responsible for the increased irrigation water demand in Xinjiang, especially in Junggar and Tarim Basins.

### The relationship between irrigation water requirements and temperatures and cultivated land areas

**Figure 7:**
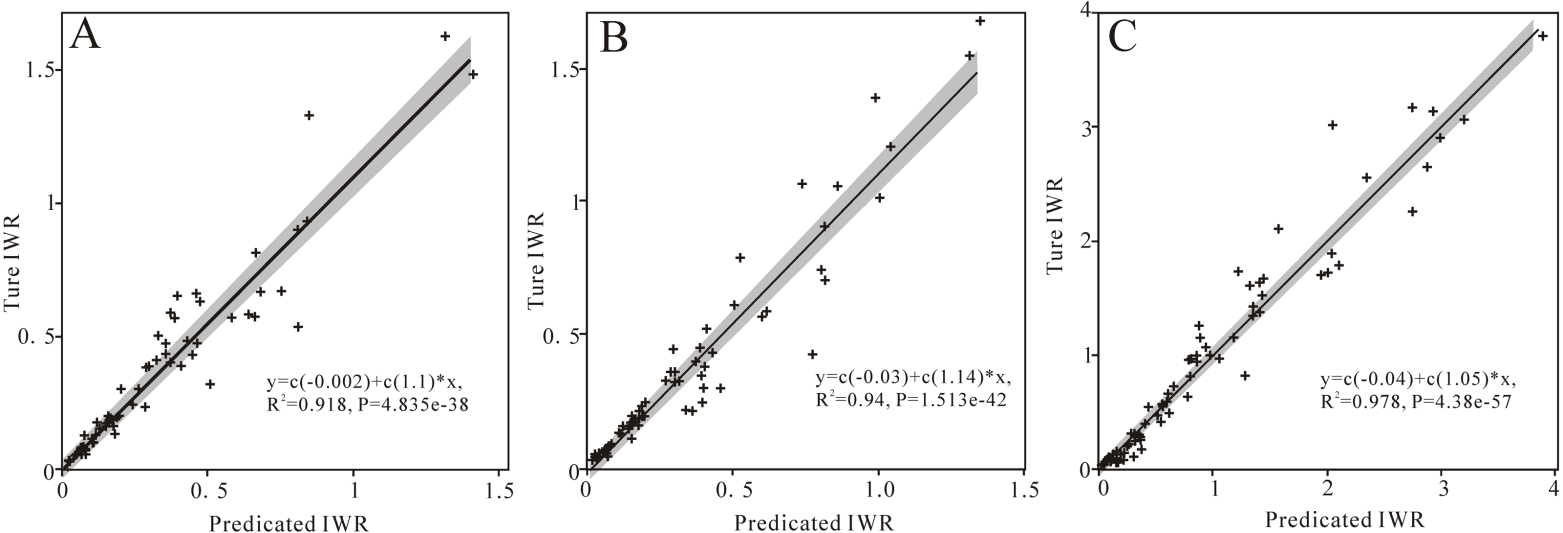
Model performance using scatter plots of predicted IWR and calculated IWR for wheat (A), corn (B), and cotton (C).

We found that changes in IWR were mainly influenced by crop growing areas. Furthermore, we need to determine the contributions of these factors (temperatures and arable land areas) to IWR via random forest modeling with an expectation for better water allocation guidance for the sustainable development of agriculture. The results showed that IWR validations, *R*^2^ and RMSE, were 0.96 and 0.221 for cotton, 0.96 and 0.23 for wheat, and 0.95 and 0.12 for corn, respectively. We evaluated the model performance using scatterplots of predicted and calculated IWR (Fig. 7), the line in this figure is a linear fit to the scatter, and the formula and the *R*^2^ value for this fitting line are shown in the figure. The results showed that the variable importance of cultivated land areas of cotton and temperature were 72.17% and 19.28%, respectively; those of wheat were 89.52% and 22.96%, respectively; and those of corn were 98.67% and 33.91%, respectively (Table 2). These results revealed that the contribution rate of arable land to IWR was greatest, which was consistent with the increase in IWR with the expansions of the planting areas of wheat, corn and cotton from 1995 to 2017.

**Table 2:** Modeling, verification, and prediction accuracy evaluation index using random forest regression.

	***R*^**2**^**	**RMSE**	**MAE**	**Var explained**	**Mean of squared residuals**	**Importance (%) arable land temperature**	
Wheat	0.918	0.106	0.06	0.893	0.013	98.67	33.91
Corn	0.940	0.118	0.068	0.866	0.020	89.52	22.96
Cotton	0.978	0.214	0.133	0.942	0.087	72.17	19.28

### Water balance in different basins

We analyzed the water supply and demand status of water among four basins in 2014 and 2015 because the maximum IWR occurred in these two years. The total water consumption of Xinjiang was 39.21 billion m^3^ with an average irrigation efficiency of 68% (Table 1). Due to the differences in climate change and management modes, the irrigation efficiency was different in four basins of Xinjiang (Table 1). The largest irrigation efficiency (∼79.30%) occurs in Karamay, while the lowest irrigation efficiency (∼60.40%) takes place in Kezhou.

**Figure 8:**
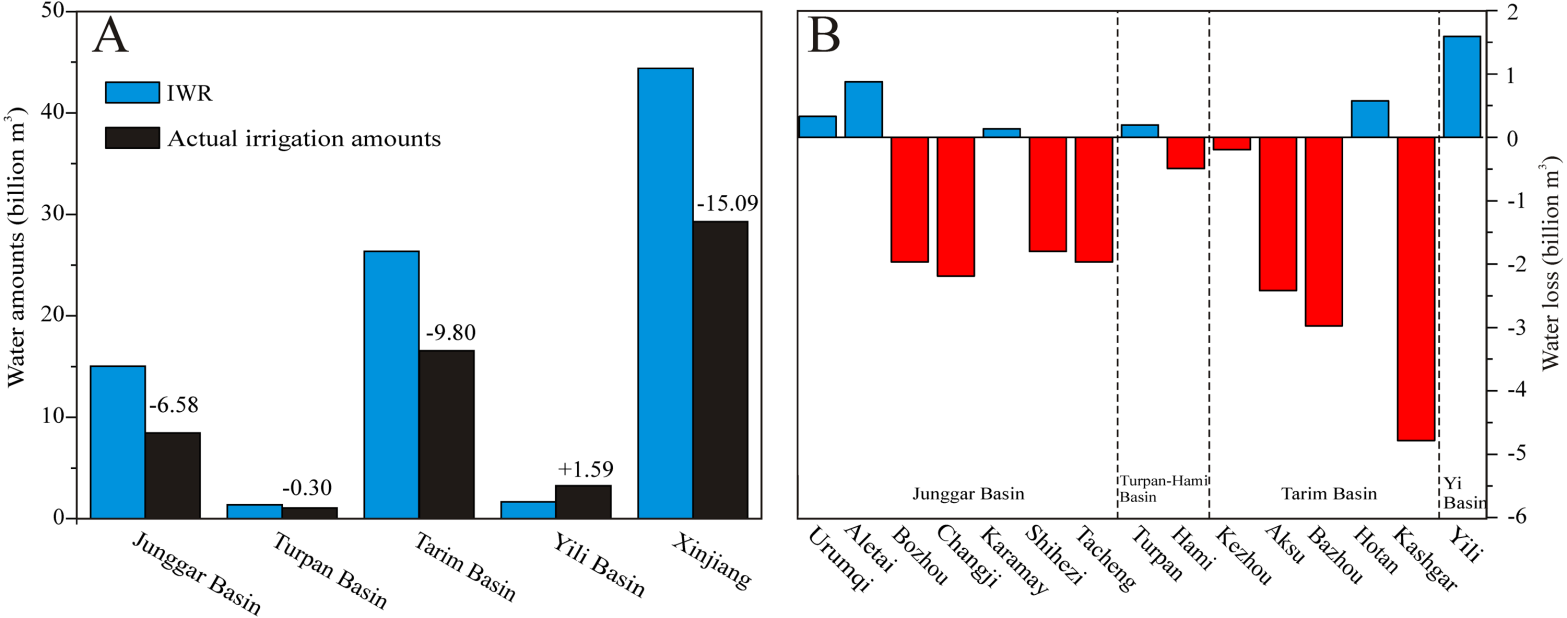
Changes in IWR and actual irrigation amounts (A) and conditions of water scarcity (B) in the different prefectures of Xinjiang in 2014.

The actual irrigation water amount in 2014 was largest in Tarim Basin with a value of 16.56 billion m^3^, and the second largest actual irrigation water amount was in Jungar Basin with 8.43 billion m^3^. The actual irrigation water amounts of other two basins (Yili Basin and Turpan-Hami Basin) were 3.26 billion m^3^ and 1.06 billion m^3^, respectively. Combined with the aforementioned analysis of IWR in four basins of Xinjiang, we found that most regions are still facing serious water scarcities (Fig. 8A). Being consistent with changes in actual irrigation water amounts, the regions with high irrigation water levels correspond to the conditions of severe water scarcity in Tarim and Junggar Basins. The levels of water scarcity in Tarim Basin were larger than those in Junggar Basin, being −9.80 billion m^3^ and −6.58 billion m^3^, respectively. The Yili Basin faces no water scarcity and the amount of the water surplus was 1.67 billion m^3^, while a slight water scarcity of −0.30 billion m^3^ was seen for Turpan-Hami Basin. Overall, the total amount of the water resource shortage in Xinjiang region for 2014 was −15.01 billion m^3^.

In Junggar Basin, only three prefectures (Urumqi, Aletai and Karamay) experienced conditions of water surplus, while the others suffered from serious water scarcity, including Bozhou, Changji, Shihezi, Karamay and Tacheng. Among these prefectures, the prefecture with the most severe water shortage was Changji (−2.19 billion m^3^), and the second most serious shortages were seen in Bozhou and Tacheng (both −1.97 billion m^3^). In Tarim Basin, only Hotan experienced no water shortage due to the abundant river resources from the Kunlun Mountains. The most severe water shortages were in Kashgar (−4.79 billion m^3^), and the second most severe shortage was in Bazhou (−2.98 billion m^3^). The third most severe shortage was in Aksu (−2.42 billion m^3^). In Turpan-Hami Basin, no water shortages occurred in Turpan. The prefectures suffering from serious water scarcity (e.g., Kashgar, Bazhou, Aksu, Tacheng, and Changji) were impacted by the expansion of crop acreages. The expansions of cotton acreage were mainly responsible for the increased water irrigation demands in Kashgar, Bazhou and Aksu within Tarim Basin. In Junggar Basin, the expansion of cotton and corn acreage was mainly responsible for increased irrigation water usage in Changji and Tacheng.

**Figure 9:**
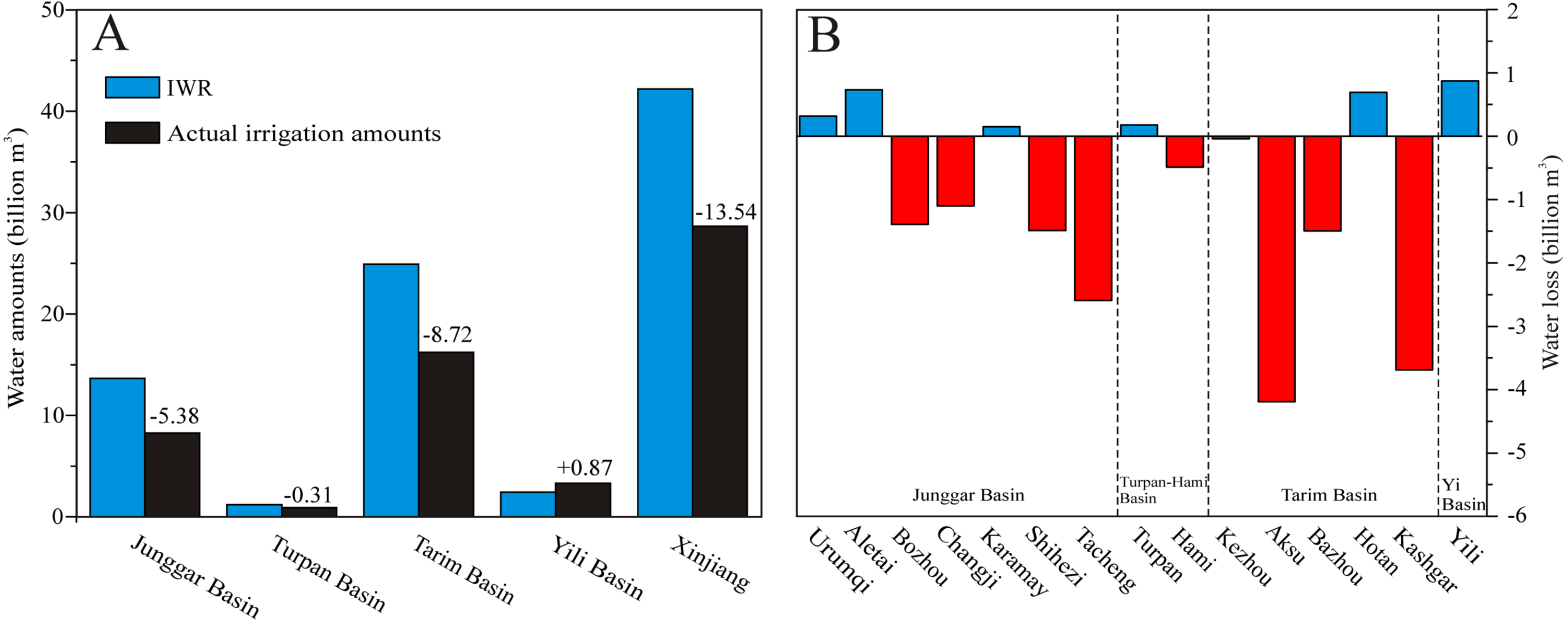
Changes in IWR and actual irrigation amounts (A) and conditions of water scarcity (B) in the different prefectures of Xinjiang in 2015.

Compared with conditions of water scarcity in four basins in 2014, the total IWR decreased slightly in 2015 due to reductions in growing areas for crops (Fig. 9A). However, most prefectures were still facing serious water scarcities (Fig. 9B). Because of changes in the planting structure of crops, the prefecture experiencing the most serious water scarcity within Tarim Basin was Aksu, with a shortage of −4.18 billion m^3^ followed by Kashgar with a shortage of −3.69 billion m^3^. In Junggar Basin, Tacheng was facing the greatest water scarcity of −2.60 billion m^3^, followed by Bozhou with a shortage of −1.39 billion m^3^. According to these analyses of water scarcity in 2014 and 2015, these conditions of long-term water scarcity led to decrease in crop production and caused degradation of the ecology in the oasis areas and for the rivers downstream, including salt accumulations in agricultural lands (Hu et al., 2013) and degradation of riparian vegetation (Chen et al., 2012). Combined with the results of Shen et al. (2013), we conclude that the levels of water resource deficiency in Xinjiang were consistently increasing under the conditions of a warming climate, especially in Kashgar and Aksu within Tarim Basin and in Tacheng, Bozhou and Changji within Junggar Basin.

## Discussions

The water used for agricultural irrigation in Xinjiang is considered making up 94.7% of its total water consumption (Xinjiang Water Resources Bulletin, 2014, 2015). Therefore, accurate estimation of IWR is urgent and necessary for scientific allocation of limited water resources, especially in arid region like Xinjiang Province. Previous studies have documented IWR in Junggar and Tarim Basins in different years. An increment of IWR can be observed from these studies which might due to cropland expansion (Shen et al., 2013; Wang et al., 2019). In comparison with their results, IWR estimated by our approach is smaller. This discrepancy in IWR lies in those key parameters involving crop selection, crop coefficient and irrigation efficiency. Considering the importance of those parameters in improving our results both in spatial and temporal scale, many relevant studies have been referenced for crop selection and the determination of crop coefficients.

It should be noted that standard-condition ET_*c*_ is the evapotranspiration from disease-free and well-fertilized crops, which are grown in large fields with optimum soil water conditions and full production under given climatic conditions (Allen et al., 1998). However, the actual crop evapotranspiration may smaller than ET_*c*_ due to non-optimal conditions such as the presence of diseases, soil salinity, low soil fertility, water shortage or waterlogging. In this study, the underlying assumptions were made: disease-free crops, well-fertilized soil and full-production. However, the optimum soil water conditions in arid regions including Xinjiang may only resemble well-water conditions during irrigation and shortly after precipitation. As the days related to optimum soil water conditions passed by, the ET_*c*_ shall decrease as the decrease of soil water content until the advent of next irrigation (Allen et al., 1998). In addition, the whole province is featured by a dry climate system and an irrigation dominant agricultural system.

According to our comparison between estimated IWR and actual irrigation water amount in 2014 and 2015 in Xinjiang, our results might be critical in three aspects: (1) over-irrigated (i.e., water surplus) prefectures (e.g., Yili Basin and Altai prefecture) should reduce actual irrigation water. (2) Serve as a reference for water reallocation. Due to the results that water resources are not evenly distributed in space, the allocation of water resources should be rationally planned to transfer water from prefectures with surplus water resources to neighboring prefectures suffering water shortage. For example, excess water in Hotan can be transferred to the nearby Kashgar to reduce the situations of water shortage. (3) Lay a foundation for studies related to agricultural water estimation in the big background of global climate change.

Furthermore, Xinjiang experienced a warming and wetting climate in the past decades (Li et al., 2013; Luo et al., 2019; Wang & Qin, 2017) which might extend to the future (Che et al., 2018; Huang et al., 2018; Li et al., 2017a). The available water and agricultural water demand in arid Xinjiang under the future climate change scenario could increase due to the rising in temperature (Guo & Shen, 2016; Yang et al., 2014). To better cope with future climate change in protecting oases ecosystem and meeting the demand of residents, measures with great wisdom including large-scale application of water-saving irrigation technologies (e.g., drip irrigation and micro-spray irrigation) readjustment of the planting structure of crops, as well as the implement of water price management (i.e., the reasonable use of crop prices can control the planting area of crops) (Chartzoulakis & Bertaki, 2015) should be taken in those extremely water-scarce prefectures. Further works will need to classify the degree of water shortage and to compare the IWR of different water shortage areas (Fu et al., 2019; Han et al., 2018; Wang et al., 2019). In addition, considering the refined IWR for each watershed, extracting crop growing area and forecasting future water irrigation requirement could be further needed.

## Conclusions

Based on the PM-FAO algorithm, we estimated regional irrigation water requirements (IWR) and water balance in Xinjiang during 1995–2017. Results suggested that the total IWR was 38.99 billion m^3^ in 2017, an increase of 24.87 billion m^3^ compared to 1995. From the perspective of crops, IWR of cotton reached 26.39 billion m^3^ in 2017, accounting for 67.68% of the total water consumption.

For changes of IWR in four basins, the fastest increasing rate was in Tarim Basin, which was attributable to the increase in water requirement for cotton. Compared our estimated IWR and actual irrigation in Xinjiang in 2014, the amount of water shortage in 2014 had reached −15.09 billion m^3^ (−9.80 billion m^3^ in Tarim Basin and −6.58 billion m^3^ in Junggar Basin). The main reason for the increase in IWR could be an extensive cotton-planting project in Xinjiang.

Considering the change of cultivated land area and the response of irrigation water to temperature, the planting area of three main crops (wheat, corn and cotton) were more sensitive to IWR than temperature factor.

##  Supplemental Information

10.7717/peerj.8243/supp-1 Data S1 Raw data

## Additional Information and Declarations

### Data Availability

The following information was supplied regarding data availability: Meteorological datasets for Xinjiang are available at the China Meteorological Administration (http://data.cma.cn/). Land use/cover data are available at the National Land Use/Cover Dataset (NLCD) and at the Earth System Science Data Sharing Infrastructure of the Chinese Academy of Sciences (http://www.resdc.cn). Socioeconomic data are available at the statistics bureau of Xinjiang Uygur Autonomous Region (http://www.xjtj.gov.cn/) and the China Statistical Yearbooks Database (http://tongji.cnki.net/kns55/navi/HomePage.aspx?id=N2019010150&name=YXJTJ&floor=1). Raw data are available as a Supplemental File.
